# Profiling bacterial diversity in a limestone cave of the western Loess Plateau of China

**DOI:** 10.3389/fmicb.2015.00244

**Published:** 2015-03-30

**Authors:** Yucheng Wu, Liangcheng Tan, Wuxing Liu, Baozhan Wang, Jianjun Wang, Yanjun Cai, Xiangui Lin

**Affiliations:** ^1^State Key Laboratory of Soil and Sustainable Agriculture, Institute of Soil Science – Chinese Academy of Sciences, NanjingChina; ^2^State Key Laboratory of Loess and Quaternary Geology, Institute of Earth Environment – Chinese Academy of Sciences, Xi’anChina; ^3^Joint Center for Global Change Studies, BeijingChina; ^4^Key Laboratory of Soil Environment and Pollution Remediation, Institute of Soil Science – Chinese Academy of Sciences, NanjingChina; ^5^State Key Laboratory of Lake Science and Environment, Nanjing Institute of Geography and Limnology – Chinese Academy of Sciences, NanjingChina

**Keywords:** bacterial diversity, cave, rock surface, pyrosequencing, elemental composition, 16S rRNA

## Abstract

Bacteria and archaea sustain subsurface cave ecosystems by dominating primary production and fueling biogeochemical cyclings, despite the permanent darkness and shortage of nutrients. However, the heterogeneity and underlying mechanism of microbial diversity in caves, in particular those well connect to surface environment are largely unexplored. In this study, we examined the bacterial abundance and composition in Jinjia Cave, a small and shallow limestone cave located on the western Loess Plateau of China, by enumerating and pyrosequencing small subunit rRNA genes. The results clearly reveal the contrasting bacterial community compositions in relation to cave habitat types, i.e., rock wall deposit, aquatic sediment, and sinkhole soil, which are differentially connected to the surface environment. The deposits on the cave walls were dominated by putative cave-specific bacterial lineages within the γ-Proteobacteria or Actinobacteria that are routinely found on cave rocks around the world. In addition, sequence identity with known functional groups suggests enrichments of chemolithotrophic bacteria potentially involved in autotrophic C fixation and inorganic N transformation on rock surfaces. By contrast, bacterial communities in aquatic sediments were more closely related to those in the overlying soils. This is consistent with the similarity in elemental composition between the cave sediment and the overlying soil, implicating the influence of mineral chemistry on cave microhabitat and bacterial composition. These findings provide compelling molecular evidence of the bacterial community heterogeneity in an East Asian cave, which might be controlled by both subsurface and surface environments.

## Introduction

Most biological communities are dependent on the energy and carbon fixation of photosynthesis. However, perpetual darkness prevents the colonization of phototrophs in subterranean cave environments. Limited energy and nutrients can enter caves via entrance, sinkholes, underground hydrology, and drip waters (Barton and Jurado, 2007), and the aphotic and oligotrophic environments only allow for the survival and functioning of species adapted to the oligotrophic conditions. This is clarified by the dominance of microbial chemoautotrophic production in some cave ecosystems (Sarbu et al., 1996; Chen et al., 2009), which fixes carbon and imports energy into cave food web.

Bacteria and archaea constitute the vast majority of biodiversity in caves and are ubiquitous in various cave habitats such as soils, sediments, stream waters, and rock surfaces (Engel et al., 2004; Barton and Jurado, 2007). Many known bacterial phyla have been detected in cave environments by sequencing of 16S rRNA genes (Engel et al., 2004; Barton et al., 2007; Ortiz et al., 2013b), greatly advancing our understanding of bacterial diversity since its introduction to microbial ecology (Roesch et al., 2007). For example, the dominant taxa on cave walls are largely affiliated with a few phyla such as Proteobacteria, Acidobacteria, and Actinobacteria (Barton et al., 2007; Pašić et al., 2010; Cuezva et al., 2012). Bacterial richness in cave sediments could be comparable to that in overlying soils (Ortiz et al., 2013b), but the rock surfaces are normally colonized by the lowest diversity natural microbial communities (Macalady et al., 2007; Yang et al., 2011). By comparison of geographically distinct caves, which mostly locate in Europe and America, it was supposed that rock surfaces could be colonized by common phylotypes that were rarely found in other habitats (Porca et al., 2012), suggesting the presence of specific cave bacterial lineages. However, whether they are present in caves in other area, e.g., East Asia merits further study.

Mounting evidence indicates that microbes may sustain cave ecosystems by dominating primary production and fueling biogeochemical cyclings. This has been revealed in some deep caves that are well isolated from the surface environment, including the Movile cave (Romania; Sarbu et al., 1996), the Frasassi cave (Italy; Desai et al., 2013), and the Kartchner Caverns (US; Ortiz et al., 2013a). In these caves, chemoautotrophic microbes are largely responsible for CO_2_ fixation and potentially participate in inorganic nitrogen (Desai et al., 2013; Tetu et al., 2013) and sulfur transformation (Chen et al., 2009). Moreover, interactions between microorganisms and rock may contribute to speleogenesis, the process of cave development that was previously viewed as abiotic and often attributed to the erosion of bedrock by water containing dissolved CO_2_ (Engel et al., 2004). For example, in sulfidic caves, the microbial oxidation of H_2_S produces sulfuric acid, which reacts with carbonate and causes rock dissolution (Engel et al., 2004; Macalady et al., 2007). Bacteria are able to modify rock surfaces through the oxidation of some metal elements [e.g., Fe(II) and Mn(II)], which causes ferromanganese depositing on cave walls (Carmichael et al., 2013).

Cave microbial communities are often highly variable dependent on the microhabitats. Significantly different bacterial diversity and composition were observed on rock walls within one single cave, possibly related to the host rock geochemistry (Barton et al., 2007). Nutrient inputs and disturbances may also contribute to cave microbial diversity. Organic matter and microbes could be carried into caves by air current, seepage water (Shabarova et al., 2013) and animal and human activities. The latter is a serious issue especially for caves open for tourism, which may introduce unwanted species and cause deterioration of paleolithic paintings (Ikner et al., 2007; Diaz-Herraiz et al., 2013). As such, cave bacterial diversity could be shaped by both interior (e.g., the rock geochemistry) and exterior (natural or anthropogenic) environments, and the influence of the latter is likely dependent on the cave’s connectivity to outside world. However, the heterogeneity and mechanism of microbial diversity inside of caves, in particular in those easily exposed to the surface are still poorly understood.

In this study, we focused on the bacterial communities in Jinjia Cave, a limestone cave in the semi-humid western Loess Plateau of China. The cave is short and shallow in contrasting to those studied previously, and was supposed to be influenced by the surface due to the limited depth. By using next generation sequencing of 16S rRNA genes, we profiled the bacterial communities colonizing different habitats including rock wall deposits, sediments in small pool and sinkhole, and overlying soils. Meanwhile, the microhabitat characteristics were examined by a detailed analysis of the elemental composition of the sample. The aims of the study were therefore to explore the bacterial heterogeneity within a single cave in relation to habitat type and to evaluate the linkage between inside and outside bacterial communities.

## Materials and Methods

### Cave Description and Sampling

**FIGURE 1:**
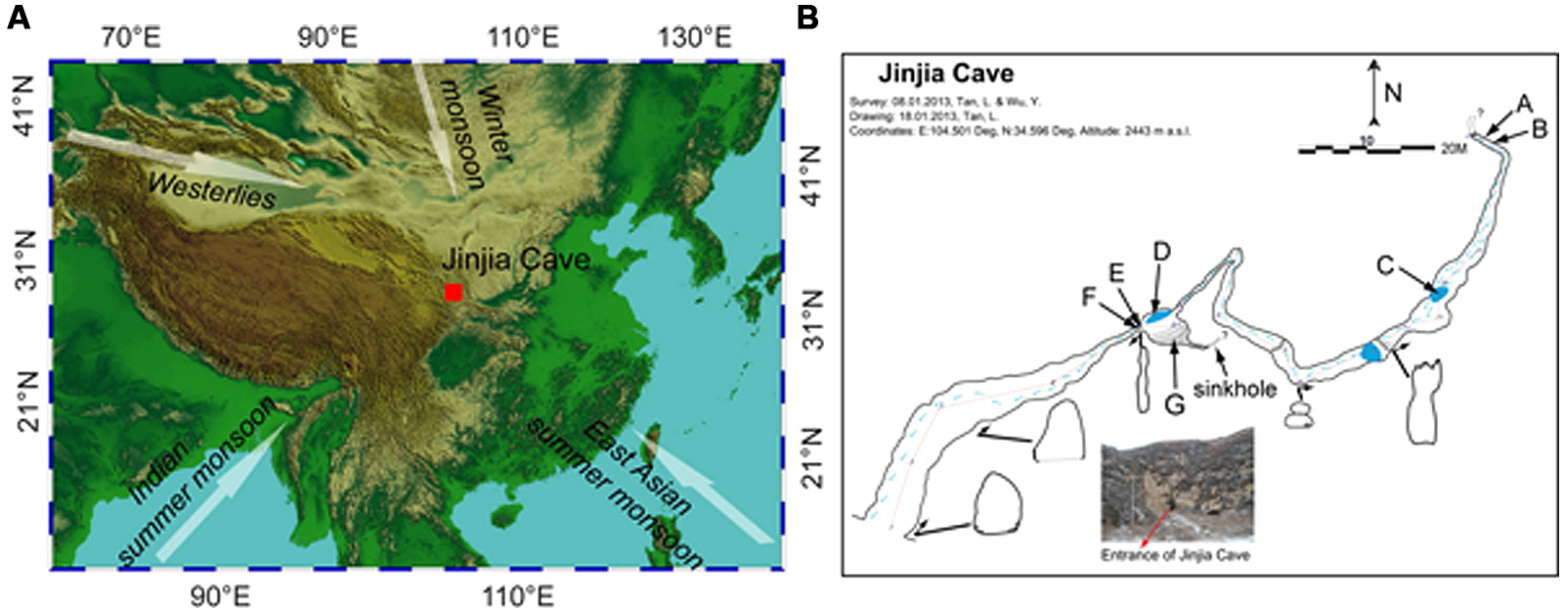
**Location (A) and plan view (B) of Jinjia Cave**. Arrows in **(A)** indicate the directions of the east Asian summer monsoon, Indian summer monsoon, east Asian winter monsoon, and westerlies. The letters A and B (cave wall deposit), C and D (pool sediment), E and F (cave wall deposit), and G (sediment) in **(B)** mark the sampling sites inside the cave. Samples H, I, and J were from soils overlying the cave and were not shown in **(B)**.

Jinjia Cave (34°35.80′N, 104°30.05′E, 2443 m a.s.l.) is located approximately 25 km southeast of Zhang County, east Gansu Province, China (**Figure 1A**). This area is in the western Loess Plateau, with an annual precipitation of ~440 mm and average temperature of ~7.4°C. Most of the rainfall (>80%) occurred in the monsoon season. Modern vegetation overlying the cave was composed of temperate scrub and grass (Tan, 2008).

The cave, formed of Permian limestone, has an entrance of ~6 m × 7 m with ~200 m in length and 40 m-thick ceiling rock. The underground river was dry upon sampling, but a few small and shallow pools with a water depth of ~5–10 cm were present (**Figure 1B**). The cave was not open for tourism, and hence was less exposed to human activity. When sampling from the entrance to the end of the cave in January 2013, the temperature increased from -5.5°C at the entrance to 7°C at the deepest passage.

To avoid the possible anthropogenic influence at the entrance and twilight zones, seven samples from the dark zone of the cave were collected, including four deposits on cave wall and two sediments in pools and in the soil at the bottom of a sinkhole. Among them, two cave wall deposits (E/F) and one sediment (G) were collected in the chamber ~60 m from the main entrance (outer chamber), and the other two deposits (A/B) were obtained from the accessible deepest part of the cave (inner passage, **Figure 1B**). During sampling, the loose minerals were collected by scraping the rock surface with a sterile spatula. Two sediment samples (C/D) were collected from pools in the outer chamber and a passage approximately 3/4 of the way into the cave, respectively. In addition, three overlying soils (H/I/J) were also collected. It should be noted that sample G and H were located on the bottom and the top of a small sinkhole, respectively. All the samples were put in sterile tubes, transported under the refrigeration to the laboratory and stored at -20°C until use.

### Elemental Analysis

The samples were freeze-dried, finely ground to 200 mesh size (<74 μm) and homogenized in an agate mortar. Five grams of the powdered sample was compressed into a thin compact disk. Concentrations of major (e.g., Si, Al, and Ca) and trace elements (e.g., Rb, Sr, and Ba) were analyzed using a Philips PW4400 X-ray fluorescence (XRF) spectrometer at the Institute of Earth Environment – Chinese Academy of Sciences (Sun et al., 2008). Calibration was performed with Chinese national soil reference samples. Analytical precision was better than 1% for major elements and better than 5% for trace elements. The Student’s *t*-test and principal component analysis (PCA) were carried out with SPSS 13.0 to inform the difference between rock deposit and soil/sediment samples and the similarity in sample elemental composition, respectively.

### DNA Extraction and Quantitative PCR (q-PCR) of Bacterial 16S rRNA Genes

DNA was extracted from approximately 0.5 g samples (fresh weight) with the FastDNA SPIN Kit for Soil (MP Biomedicals) following the manufacturer’s instructions. Ten-fold diluted DNA did not significantly inhibit the PCR reaction by calculating the q-PCR amplification efficiency of bacterial 16S rRNA gene with serial dilutions of extracts, therefore was used in all downstream molecular analysis.

Quantitative-PCR was performed on an iCycler instrument (Bio-Rad). The primer sets of 515f and 907r (Stahl and Amann, 1991; Muyzer et al., 1995) were used for the quantification of bacterial 16S rRNA genes with the SYBR green-based reactions performed in triplicate for each sample. The 20-μL reaction mixture contained 10-μL TransStart Green qPCR SuperMix (Transgen, Beijing, China), 0.2 μmol L^-1^ of each primer, and 2-μL template DNA. Reactions were run with 3 min at 95°C, 40 cycles of 15 s at 95°C, 30 s at 55°C, 30 s at 72°C, following by a plate read. The amplification specificity was checked by melting curve analysis and electrophoresis. The q-PCR standards were generated using plasmid DNA from one clone containing bacterial 16S rRNA gene. A dilution series of the standard template across seven orders of magnitude (10^1^–10^7^) was used per assay. The control was run with water as the template instead of DNA extract. The q-PCR amplification efficiency for the standards was 97.7% with *r*^2^-value of 0.997.

### 454 Pyrosequencing

Pyrosequencing of 16S rRNA genes was performed on a Roche 454 GS FLX Titanium sequencer (Roche Diagnostics Corporation, Branford, CT, USA) as previously described (Wu et al., 2013). Briefly, the V4 region of the 16S rRNA gene was amplified from all samples with the tagged 515f and 907r primers. The triplicate PCR amplicons for each sample were pooled, gel purified, and combined in equimolar ratios into a single tube for pyrosequencing analysis. Sample information and sequences were deposited in the Sequence Read Archive of NCBI under accession number PRJNA268982.

### Sequence Analysis and Community Comparison

Analysis of 454 pyrosequencing data was conducted using the Mothur software v1.30.2^1^ (Schloss et al., 2009) combined with RDP II^2^ for taxonomic identification. All reads obtained were processed by removing tags and primer, with only reads with an average quality score above 30 and read lengths above 250 nt deemed acceptable. The trimmed sequences were aligned against the SILVA bacterial 16S rRNA gene databases using the Needleman algorithm. Chimeric sequences were identified and removed using the implementation of Chimera-uchime (Edgar et al., 2011). Potential chloroplasts and mitochondria sequences were identified and discarded by an assignment against respective sequence references. The high quality sequences were aligned to generate a distance matrix and clustering with the average neighbor algorithm. Representative sequences for each operational taxonomic unit (OTU), as defined by a 97% sequence identity, were obtained for further taxonomic analysis. Taxonomic placement of each OTU was carried out with RDP Classifier (Wang et al., 2007) with a confidence threshold of >80% selected.

Operational taxonomic unit richness and Shannon and Simpson diversity of each library were calculated at the cutoff of 0.03. Principal coordinate analysis (PCoA) was performed based on Bray–Curtis dissimilarity distances between the libraries with Mothur. Maximum likelihood (M-L) trees were calculated based on the Jukes–Cantor correction with Mega version 5.2 (Tamura et al., 2011) with representative cave sequences and the closest relatives deposited in the Genbank database. Bootstrap support was calculated (1000 replications).

## Results

### Elemental Compositions

The major and trace element concentrations of cave wall deposits, sinkhole and surface soils, and one limestone bedrock sample are shown in **Table 1**. PCA analysis indicates that the elemental compositions of deposits on the cave wall (A/B/E/F) showed broad similarities with that of the limestone bedrock (**Figure S1**). In contrast, the elemental composition of sinkhole soil (G) was highly similar to that of the soil overlying the cave (H). Significant enrichments (*P* < 0.05 or 0.01, *t*-test) of Ca and deficits of Na, Al, Si, K, Ti, and Rb in deposits from the cave walls and limestone bedrock were in contrast to those of the sinkhole soil. Notably, Mn and Fe in some deposits were especially enriched. For example, Fe in sample A was by far the most abundant element (51.9%) and was more than 5-fold higher than in limestone bedrock (9.06%), while Mn in B/F (>15%) was approximately three times higher than in bedrock (4.16%).

**Table 1:** Major and trace element concentrations of samples collected inside in and outside Jinjia Cave.

**Sample type**	**Sample ID**	Na* (%)	**Mg** (%)	Al** (%)	Si** (%)	**P** (‰)	K** (%)	Ca** (%)	**Sc** (ppm)	Ti* (‰)	**V (ppm)**	**Cr (ppm)**	**Mn (%)**	**Fe (%)**	**Co (ppm)**
Cave wall deposits	A	0.30	0.50	0.62	1.68	9.21	0.07	6.56	104	0.36	79.7	24.2	0.30	51.9	49.1
	B	0.61	0.77	1.62	6.16	1.67	0.45	5.02	0.79	0.20	185	22.1	15.0	17.0	67.6
	E	0.99	1.25	3.60	9.84	2.16	1.09	6.91	6.51	2.97	218	66.5	10.9	12.0	75.7
	F	0.59	0.70	1.53	2.96	4.46	0.30	6.24	4.74	0.33	172	59.3	15.4	32.1	16.6
Sediment	G	1.36	1.06	5.84	28.4	0.74	1.85	0.97	11.9	4.06	138	75.5	0.09	3.79	19.9
Surface soil	H	1.24	1.09	6.06	28.5	0.60	1.97	0.76	12.4	3.97	136	71.9	0.08	3.74	15.9
Limestone bedrock	–	0.23	0.35	1.00	2.15	1.44	0.20	28.7	9.33	0.52	139	14.8	4.16	9.06	29.4

**Sample type**	**Sample ID**	**Ni (ppm)**	**Cu (ppm)**	**Zn (ppm)**	**Ga%% (ppm)**	**As (ppm)**	**Rb%% (ppm)**	**Sr (ppm)**	**Y (ppm)**	Zr* (ppm)	Nb* (ppm)	**Ba** (‰)	Hf* (ppm)	**Pb (ppm)**	**CO_3_^-^** (%)

Cave wall deposits	A	<10	<10	<10	2.80	622	10.0	300	7.44	26.1	2.03	0.18	0.73	12.4	36.8
	B	181	76.2	85.2	0.42	117	15.0	990	32.1	83.8	7.49	11.6	3.95	29.7	51.7
	E	141	70.5	149	6.89	105	45.3	330	48.6	189	11.7	5.59	5.78	36.1	51.8
	F	223	<10	17.9	3.30	430	15.5	262	36.1	67.6	6.68	4.29	0.24	30.6	39.6
Sediment	G	35.5	31.6	89.9	17.0	19.0	110	144	30.6	316	16.4	0.48	9.68	39.8	55.9
Surface soil	H	34.9	30.5	90.6	17.6	15.9	119	133	27.5	240	15.8	0.50	7.71	27.9	55.9
Limestone bedrock	–	35.6	<10	9.97	3.47	139	10.9	250	11.2	55.5	4.28	1.68	0.60	11.6	53.6

* and ** represents the values for cave wall deposits and sediment/soil that are significantly different (P < 0.05 or 0.01, respectively). A bedrock sample was not included in the t-test.

### q-PCR of Bacterial 16S rRNA Genes

**FIGURE 2:**
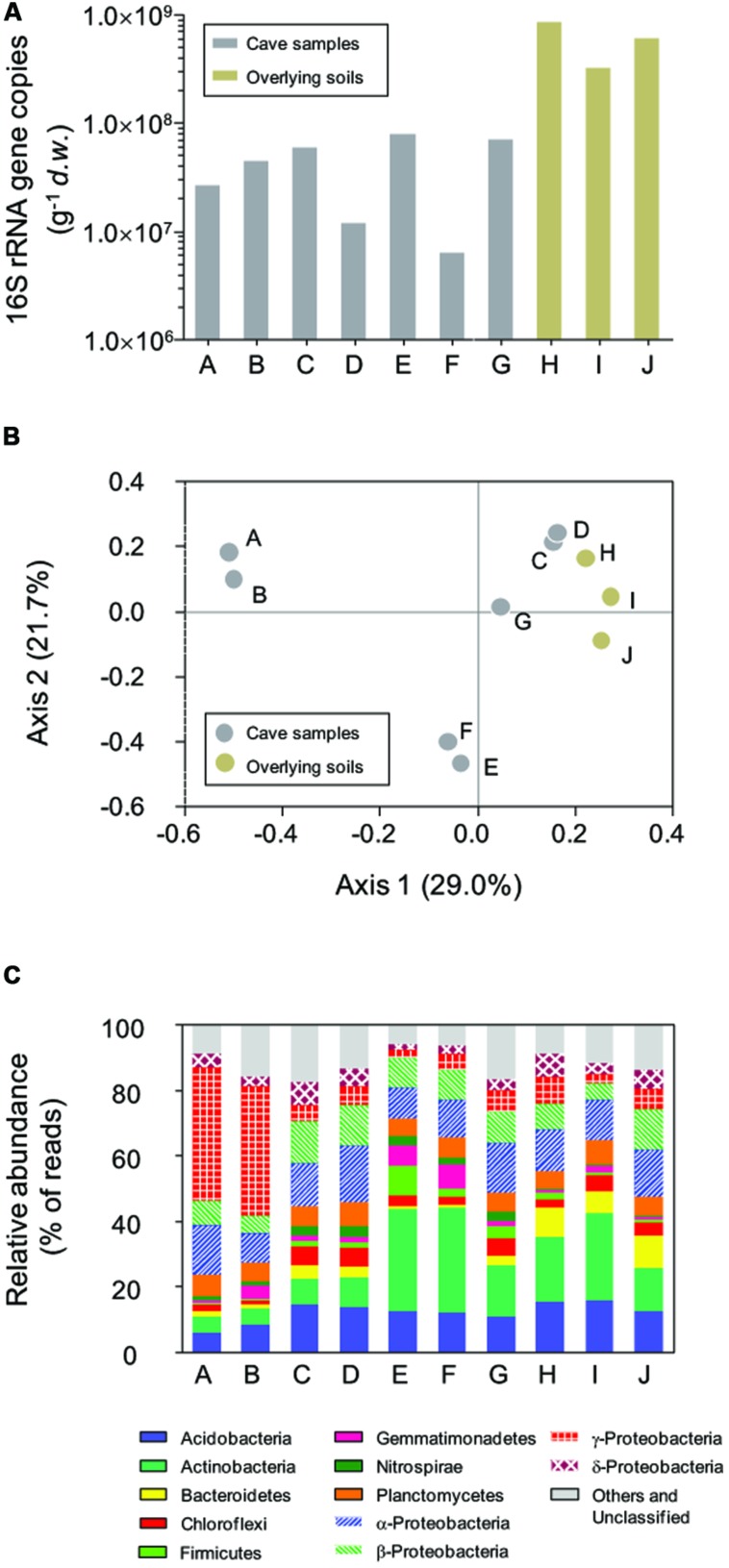
**Abundance of bacterial 16S rRNA genes (A), principal coordinate analysis (PCoA) plot based on Bray–Curtis distances (0.03 cutoff) between samples (B), and the relative abundance of dominant phyla in Jinjia Cave and related surface soils (C)**. Samples A, B, E, and F are from cave wall deposits, C and D are from pool sediments, G is a sinkhole soil and H–J are overlying soils.

Bacterial 16S rRNA genes were recovered from all samples with q-PCR (**Figure 2A**), with a high variation in copy number across two orders of magnitude observed. In the surface soils, 16S rRNA gene copies ranged from 3.18 × 10^8^ to 8.34 × 10^8^ g^-1^ *d.w.*, which were 1–2 orders of magnitude more than for samples inside in the cave. The highest 16S rRNA gene abundance in sample E inside the cave was 7.82 × 10^7^ copies g^-1^ *d.w.*, approximately 12.6 times higher than the lowest copy number (6.21 × 10^6^ g^-1^) found in sample F. No sample type or location-associated abundance pattern was observed.

### Bacterial Diversity

After quality filtering and removal of chimera, chloroplasts, and mitochondria sequences, 76532 reads (representing 50.5% of the original dataset) with an average length of 368.5-bp were extracted. The library size for each sample ranged from 6265 to 10481 reads (**Table 2**), and a total of 8127 OTUs at a 97% cutoff for sequence identity were observed. Observed OTU number and diversity index calculations for each sample were based upon randomly subsampling down to the lowest reads number (i.e., 6265) to avoid bias associated with library size (Gihring et al., 2012).

**Table 2:** Summary of 454 pyrosequencing and calculated diversity indices for each sample^a^.

Sample type	Sample	No. of qualified reads	Observed OTUs	Shannon (H′)	Inverse Simpson (1/D)
Cave wall deposits	A	8750	687	4.10	9.43
	B	7848	832	4.19	7.99
	E	6280	1067	5.24	48.7
	F	10481	918	5.05	30.1
Pool sediment	C	7084	2706	7.08	407
	D	9096	2499	6.85	357
Sinkhole soil	G	7476	2120	6.46	172
	H	6854	2620	7.01	378
Surface soil	I	6410	1972	6.54	226
	J	6265	2132	6.74	284

*^a^Operational taxonomic units (OTUs), Shannon and Simpson indices are calculated at 97% sequence identity. Random subsampling down to the lowest reads number (6265) was performed to avoid bias associated with library size.*

A large variability in OTU richness among the samples was observed, with comparable values in relation to sample type or collection location. The lowest OTU number was observed on deposits on the cave wall in the inner passage (A/B, with a mean of 759.5), followed by the deposits in the outer chamber (E/F, 992.5). The sinkhole sample G was located a few meters away from sample E and F, but the OTU number was about two times higher than the latter. Despite the low bacterial abundance, the two sediment samples from the pools (C/D) had the most diverse bacterial communities, with a mean OTU number of 2602.5, which was close to surface soils (H/I/J, 2241.3) and approximately threefold higher than deposit samples A and B. Such a diversity pattern was confirmed by the Shannon and Inverse Simpson Indices calculation (**Table 2**). Overall, the diversity in soil and sediments was significantly higher than in rock deposits (*P* < 0.01, *t*-test).

Pairwise Bray–Curtis distance between samples was calculated based on OTU classification. PCoA analysis was performed to reveal the relatedness among bacterial communities (**Figure 2B**). The first two axes of the Bray–Curtis PCoA biplot together explained 50.7% of the total variance in the communities. All communities clustered into three major groups, with deposits on cave wall forming two independent groups by their location. The third group consisted of sediments and sinkhole soil, as well as the three surface soil samples, suggesting more similar bacterial compositions in these samples.

### Taxonomic Patterns

All 454 reads were aligned against the SILVA bacterial ribosomal RNA reference database to derive the phyla-level community taxonomic profiles (**Figure 2C**). In total, 21 phyla and 11 candidate divisions were identified from 69921 out of 76532 reads, with varied dominant phyla observed in relation to the sample type and collection site. For example, deposit samples A and B from the outer chamber were highly dominated by γ-Proteobacteria, of which the relative reads abundance was almost 40% of the libraries, while the actinobacterial reads accounted for a third of the libraries of deposit samples from inner chamber (E and F). In contrast, the relative abundances of the most dominant phyla in the other samples were low, and there were apparent differences in specific phylum between cave and surface communities. For instance, the relative abundance of phylum *Nitrospira* in the surface soils (average 0.32 ± 0.18%, *n* = 3) was much lower than in subterranean samples (2.39 ± 0.82%, *n* = 7).

**FIGURE 3:**
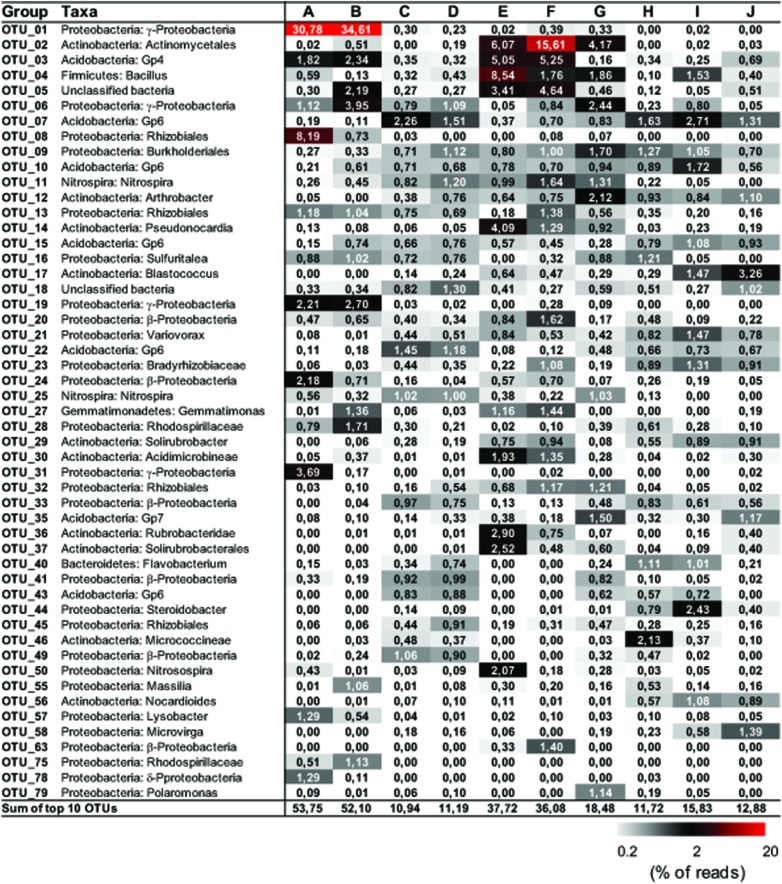
**Distribution matrix with the 10 most abundant taxa in each sample**. Abundances are based on DNA amplicons. Taxa were defined by using Classifier of RDP with 80% selected as a threshold. Numbers show the relative abundance (% of the reads) of individual operational taxonomic unit (OTU). Sequences were deposited in the NCBI Sequence Read Archive under accession number PRJNA268982.

**FIGURE 4:**
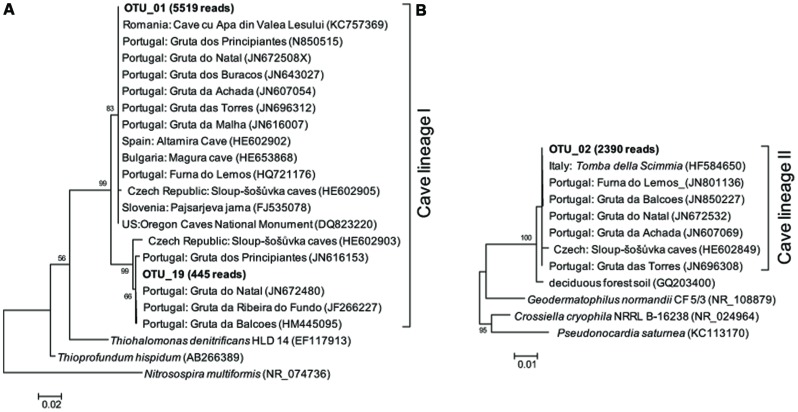
**Maximum likelihood (M-L) tree of two cave bacterial lineages, respectively, dominant in cave wall deposits A/B (A) and E/F (B)**. Sequences retrieved from the present study are shown in bold, with the number in parentheses indicating the total reads. Bootstrap values >50% are shown in the trees.

Representative sequences from the top 10 most abundant OTUs of each library were further classified with RDP (**Figure 3**). For deposit samples A and B, one OTU affiliated with γ-Proteobacteria (OTU_01) constituted 30.8 and 34.6% of the libraries, respectively. Together with the closely related OTU_19, OTU_01 represented the most dominant bacterial clade on the rock surface in the inner cave. The closest relatives in the Genbank database (96–100% sequence identity) to OTU_01 and OTU_19 were almost exclusively recovered from geographically distinct caves, constituting the cave bacterial lineage I (**Figure 4A**). The closest pure culture to OTU_01 is a chemolithoautotrophic sulfur oxidizer (*Thioprofundum hispidum*, 95% sequence identity). Surprisingly, however, the relative abundance of this clade in other two deposit samples (E/F) was minimal (<0.4%). The second most abundant OTU in deposit A (OTU_08, 8.2% of the library; **Figure 3**) was associated with environmental sequences mostly recovered from cold terrestrial habitats. The closest pure culture to OTU_08 is *Methylocella tundrae* of α-Proteobacteria (98% identity). Deposit samples E and F were predominated by Actinobacteria, Acidobacteria, and Proteobacteria (**Figure 2C**). Two Actinobacterial OTUs (OTU_02 and OTU_14) together constituted 10.2 and 16.9% of the communities, respectively, with OTU_02 routinely recovered from cave environments (cave lineage II, **Figure 4B**). Acidobacteria (OTU_03) and one unclassified phylotype (OTU_05) were also abundant in samples E and F. In addition, *Bacillus*-related sequences were only dominant in sample E (OTU_04, 8.54%).

The dominance of abundant OTUs in sinkhole soil, pool sediments, and surface soils was found to be much less than in deposits on the cave wall, as indicated by the accumulated relative abundances of the top 10 OTUs, which ranged from 10.94 to 18.48% (**Figure 3**). The most abundant taxa in pool sediments included those within subgroup 6 (Gp6) of Acidobacteria, which was relatively rare in rock deposits but constituted a major fraction in overlying soil communities (OTU_07, OTU_22, and OTU_43, **Figure 3**). Moreover, as has been revealed at the phylum level, *Nitrospira*-related OTUs (OTU_11 and 25) were relatively rare in the surface soils.

## Discussion

Jinjia Cave, like other subterranean ecosystems, is ubiquitously colonized by bacteria as indicated by the recovery of 16S rRNA genes from all samples. Assuming most bacterial genomes possess 1–7 ribosomal RNA operons (Větrovský and Baldrian, 2013), this equates to 10^6^–10^7^ bacterial cells per gram of sample, much less than that in the overlying soil but still typical of cave environments (Barton and Jurado, 2007). The low abundance of bacteria might be related to the aphotic conditions and the limited input of nutrients, which limits the proliferation of phototrophs or copiotrophs. In contrast, the bacterial richness inside the cave was more comparable to that in the surface soils, although the rock deposits had much lower OTU numbers than the cave sediments (**Table 2**). This and the diversity indices indicate the distinctness of bacterial communities on rock walls.

The rock surface might be one of the most distinct habitats in caves. The similarities in elemental compositions between the four rock wall deposits and the limestone, in particular the significant enrichment of Ca, indicates their origin from bedrock. The supplies of energy and nutrients on the rock surface are exclusively dependent on the inputs through air currents or seepage water, implying the extreme starvation, and predicting the species sorting under these conditions. Mineral chemistry may also impact the microbial community inhabiting the rock surface (Hutchens et al., 2010). Consistently, all of the four rock deposits were dominated by a few phylotypes that have been identified in geographically distinct European and American caves and may represent cave-adapted bacterial lineages. The cave lineage I within γ-Proteobacteria (**Figure 4A**) found in inner cave deposit samples A and B (32.99 and 37.31% of the reads, respectively) is likely an obligate cave clade, because the more similar sequences (>96% identity) in Genbank were exclusively recovered from caves. The OTU_01-related phylotypes have been found to be a major component of yellow mats attached to rock surfaces and was proposed as a core OTU in several caves (Porca et al., 2012). To our knowledge, this is the first evidence of the presence of cave-specific bacterial lineages in an East Asian cave. Currently there is no cultivated representative of the lineage, and the distantly related pure cultures include chemolithoautotrophic sulfur oxidizers *T. hispidum* (Mori et al., 2011) and *Thiohalomonas denitrificans* (Sorokin et al., 2007). Whether this lineage lives an obligate autotrophic and sulfur-transforming life and what the mechanism of their adaptation to oligotrophic rock surface may be both merit further study.

Rocks of the outer chamber shared less overlap with those in the inner passage, suggesting different habitat traits from the innermost passage rock walls, despite their similarity in elemental components. The most abundant bacteria in deposit samples E and F were closely related to Pseudonocardiaceae of Actinobacteria (OTU_02), which are common on subterranean rocks (Porca et al., 2012) but also present in soils. It has been suggested that organic carbon exposure may contribute to the enrichment of Pseudonocardiaceae on rock surfaces (Diaz-Herraiz et al., 2013). Consistent with this idea and in contrast to the deeper cave, the outer chamber is more easily accessible, and thus the availability of organic C might have been high due to the inputs through the main entrance or other connections such as the nearby sinkhole (**Figure 1**). Nonetheless, because of the low sequence identity of OTU_02, as well as the other abundant phylotypes to known strains, its functional traits on the rock surfaces of the inner cave are still unclear.

In addition to the enrichment of putative cave lineages, there were abundant putative chemolithoautotrophic taxa inside Jinjia Cave, such as *Nitrospira* and *Nitrosospira*, for which all cultivated representative are capable of autotrophic C fixation (Koops et al., 2006; Lücker et al., 2010). *Nitrospira* and *Nitrosospira* are involved in the two-step autotrophic nitrification, suggesting the presence of the CO_2_-fixation-coupled ammonia oxidation process, which has been supposed to sustain the primary production of some cave ecosystems (Sarbu et al., 1996; Ortiz et al., 2013a). Chen et al. (2009) also identified the activity of ammonia and sulfur-oxidizing bacteria in the chemolithotrophic Movile Cave using a stable isotope approach. As such, it is very likely that lithochemotrophy is one of the dominant bacterial life strategies in the darkness of Jinjia Cave.

Previous studies have suggested microbial roles in the formation of Mn- and Fe-rich deposits on the cave rock walls (Spilde et al., 2005; Carmichael et al., 2013). Mn-oxidizing bacteria mostly fall within Firmicutes, Proteobacteria, and Actinobacteria (Tebo et al., 2005), among which *Bacillus* has been well studied as a model Mn oxidizer (Francis and Tebo, 2002). By contrast, most iron-oxidizing bacteria are affiliated with Proteobacteria (Hedrich et al., 2011). Some *Methylocella* of α-Proteobacteria possess the capability of Fe(II) oxidation (Lu et al., 2010). Interestingly, *Bacillus*- (OTU_04) and *Methylocella*-related phylotypes (OTU_08) dominated two deposit samples in Jinjia Cave, while iron-reducing *Aciditerrimonas*-related sequences (OTU_30; Itoh et al., 2011) were also enriched in all deposit samples, implicating bacterial involvement in the ferromanganese deposit formation in Jinjia Cave. Further cultivation and metabolism analysis will be helpful for revealing the geochemical roles of these abundant and many other relatively rare bacteria.

The spatial heterogeneity in bacterial communities inside Jinjia Cave, as revealed by the broad variability of richness, composition, and dominant phylotypes, might be related to the substantial difference in cave habitats. This heterogeneity was even obvious for the same sample type (e.g., deposits A/B versus E/F; Ortiz et al., 2013b). In other cases, however, samples share common dominant phylotypes despite their considerable spatial distance. For instance, in this study, both pool sediments that are connected by underground hydrology revealed nearly identical dominant OTUs. Elemental analysis provided some clues of species sorting by microhabitats. The highly similar elemental compositions of samples G and H are not surprising (**Table 1**), because the sediment sample G very likely originated from the surface materials that could be carried into the cave via the sinkhole. This partially explains why sample G had a distinct bacterial community from the nearby rock wall deposits (E/F).

In contrast, the community overlaps between cave sediments and surface soils suggests an influence from surface environments. Due to the limited depth and length of Jinjia Cave, nutrients, energy, and microbes can constantly enter through the entrance, sinkholes, underground streams, and drip water and can thus modify microhabitat and shape microbial community. This is highlighted by the similarities in elemental and bacterial compositions between samples G and H that were associated with a small sinkhole, whereas bacterial communities in pool sediments were very likely partly controlled by underground stream and seepage waters carrying dissolved organic carbon and nutrients from the surface. Mass effect could be another factor affecting the bacterial communities in Jinjia Cave (Shabarova et al., 2013), because microbes on the surface may disperse via seepage water into the subterranean environments. This is partly supported by the fact that the pool and sinkhole sediments had more chloroplasts contamination (0.11–0.25% of each library) than rock deposits (0–0.04%), which was very likely imported from the overlying soils (0.92–1.35%). Seasonal variation in temperature within the cave might contribute to the bacterial heterogeneity too, as suggested by the difference of 12.5°C from the entrance to the deepest passage on the sampling. Overall, the external influence on cave bacterial community structure seems not to be negligible and requires further study.

## Conclusion

In this study, we profiled the bacterial communities in a short and shallow limestone cave, Jinjia Cave from western Loess Plateau of China by ribo-tag pyrosequencing. Highly heterogeneous bacterial diversity in Jinjia Cave could be related to the habitat type and might be affected by interconnection between subsurface and surface environments. The rock wall deposit communities were less diverse and dominated by possible indigenous cave bacteria, which is likely controlled by the rock surface geochemistry. Based on comparison with known functional guilds, we speculate that the bacteria on cave walls are potentially involved in CO_2_ fixation, N transformation, and speleogenesis. However, due to the absence of more biogeochemical and microbial activity information, the functions of the dominant cave bacterial lineage are still an open question.

## Conflict of Interest Statement

The authors declare that the research was conducted in the absence of any commercial or financial relationships that could be construed as a potential conflict of interest.

## Footnotes

1 http://www.mothur.org/

2 http://rdp.cme.msu.edu/

## Supplementary Material

The Supplementary Material for this article can be found online at:
