# Supplementary material for: Profiling bacterial diversity in a limestone cave of the western Loess Plateau of China
Source: Front Microbiol. 2015 Mar 30;6:244. doi: 10.3389/fmicb.2015.00244 (PMC4378288; doi:10.3389/fmicb.2015.00244)
Supplement: Supplementary file 1 [file table_1.doc]

***Supplementary Material***

**Profiling bacterial diversity in a limestone cave of the western Loess Plateau**

Yucheng Wu,^1^#*, Liangcheng Tan,^2^#* Wuxing Liu,^3^ Baozhan Wang,^1^ Jianjun Wang,^4^ Yanjun Cai,^2^ Xiangui Lin^1^

^1^ State Key Laboratory of Soil and Sustainable Agriculture, Institute of Soil Science, Chinese Academy of Sciences, Nanjing, China;

^2^ State Key Laboratory of Loess and Quaternary Geology, Institute of Earth Environment, Chinese Academy of Sciences, Xi’an, China;

^3^ Key laboratory of Soil Environment and Pollution Remediation, Institute of Soil Science, Chinese Academy of Sciences, Nanjing, China;

^4^ State Key Laboratory of Lake Science and Environment, Nanjing Institute of Geography and Limnology, Chinese Academy of Sciences, Nanjing, China

***Correspondence:**

Dr. Yucheng Wu

State Key Laboratory of Soil and Sustainable Agriculture

Institute of Soil Science, Chinese Academy of Sciences

71 East Beijing Road, Nanjing 210008, China

[ycwu@issas.ac.cn](mailto:ycwu@issas.ac.cn)

Dr. Liangcheng Tan

State Key Laboratory of Loess and Quaternary Geology

Institute of Earth Environment, Chinese Academy of Sciences

97 Yanxiang Road, Xi’an 710061, China

[tanlch@ieecas.cn](mailto:tanlch@ieecas.cn)

1. **Supplementary Figure**


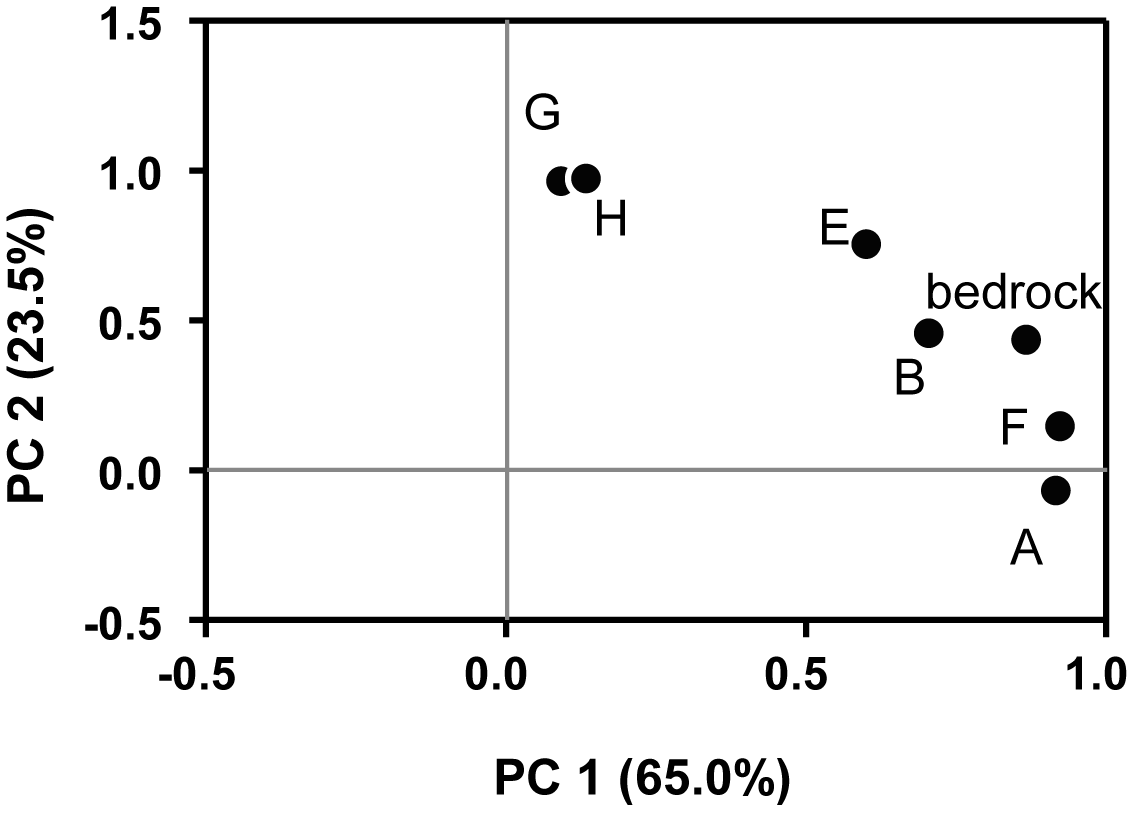


Supplementary Figure S1 PCA of elemental composition of some samples collected inside and outside Jinjia Cave.
